# Characteristics of transmission routes of COVID-19 cluster infections in Gangwon Province, Korea

**DOI:** 10.1017/S0950268821002788

**Published:** 2022-01-07

**Authors:** Chaeyun Lim, Youngju Nam, Won Sup Oh, Sugeun Ham, Eunmi Kim, Myeonggi Kim, Saerom Kim, Yeojin Kim, Seungmin Jeong

**Affiliations:** 1 Gangwon Centre for Infectious Diseases (affiliated to Korea Disease Control and Prevention Agency and Gangwon Provincial Office), Gangwon, Korea; 2 Division of Infectious Diseases, Kangwon National University Hospital, Gangwon, Korea; 3 Department of Preventive Medicine, Kangwon National University Hospital, Gangwon, Korea

**Keywords:** COVID-19, disease transmission, cluster infection

## Abstract

This study investigated the characteristics of transmission routes of COVID-19 cluster infections (⩾10 linked cases within a short period) in Gangwon Province between 22 February 2020 and 31 May 2021. Transmission routes were divided into five major categories and 35 sub-categories according to the relationship between the infector and the infectee and the location of transmission. A total of 61 clusters occurred during the study period, including 1741 confirmed cases (55.7% of all confirmed cases (*n* = 3125)). The the five major routes of transmission were as follows: ‘using (staying in) the same facility (50.7%), ‘cohabiting family members’ (23.3%), ‘social gatherings with acquaintances’ (10.8%), ‘other transmission routes’ (7.0%), and ‘social gatherings with non-cohabiting family members/relatives’ (5.5%). For transmission caused by using (staying in) the same facility, the highest number of confirmed cases was associated with churches, followed by medical institutions (inpatient), sports facilities, military bases, offices, nightlife businesses, schools, restaurants, day-care centres and kindergarten, and service businesses. Our analysis highlights specific locations with frequent transmission of infections, and transmission routes that should be targeted in situations where adherence to disease control rules is difficult.

## Introduction

A high number of cluster infections have been reported during the coronavirus disease (COVID-19) pandemic [1–3]. Cluster infections are an important determinant of the rate of COVID-19 transmission [4, 5]. A significant proportion of community cases of infection form clusters, which are closely associated with the lifestyle patterns of confirmed cases. Large-scale clusters can be formed within a short period from transmission linked to family members, colleagues, and acquaintances of the infector and/or individuals who have used/stayed the same facilities as the infector. To date, several studies have investigated the relationship between cluster infections and epidemiological characteristics of confirmed cases associated with cluster infections [6–13].

When a cluster infection occurs, many confirmed cases within a short period may lead to a shortage of medical resources, including negative-pressure beds. Moreover, a rapid increase in epidemiological investigation targets may overload local health authorities and lead to socioeconomic recession in local communities.

Given that cluster infections lead to a large increase in the total number of confirmed cases, identifying and addressing links of infection within a cluster in a timely manner are key to disease control. As such, it is necessary to elucidate the transmission routes of infection and circumstances associated with the transmission, such as the relationship between the infector and infectee as well as locations of occurrence of chains of transmission [5]. Although the characteristics of mass clusters have been reported in the literature, no study to date has analysed the transmission routes of all confirmed cluster infection cases within the entire COVID-19 infection period in a specific region. Accordingly, the present study aimed to analyse all cluster infection cases that occurred in Gangwon Province between the first confirmed case of COVID-19 on 22 February 2020 and 31 May 2021 in order to identify the transmission routes and characteristics of cluster infections.

## Methods

### Setting

Gangwon Province is an administrative district (province) with an area of 16 829.7 km^2^, a total population of 1 536 175 people, and a population density of 91.3 people/km^2^. It has sparse population density because of its mountainous terrain. Mountainous zones with altitudes higher than 100 m account for approximately 94% of total land, comprising a mix of urban and rural areas. Over 72% of the population of Gangwon Province is concentrated in urban areas located in flatlands [14].

In Gangwon Province, epidemiological investigations of confirmed COVID-19 cases were conducted as follows. Anyone suspected of having COVID-19 received a PCR test at the city/county COVID-19 screening centre. Information on confirmed positive PCR test results was relayed to each city/county public health centre. A notice containing instructions to isolate/quarantine was sent to the patient by the public health centre. Basic epidemiological investigations were conducted by phone or in person using a structured record sheet. In the basic epidemiological investigation, demographic information and the reason for undergoing a PCR test were documented. The results were registered in the COVID-19 management system of the Korea Disease Control and Prevention Agency (KDCA) in real time. To gather more detailed information about the transmission, the public health centre conducted in-depth epidemiological investigations of all confirmed patients. The in-depth epidemiological investigation verified where the patient stayed and with whom they had contact in the period between 2 days before the symptom onset date and the COVID-19 test date. If the patient did not manifest symptoms of COVID-19, the tracing was conducted from 2 days before the COVID-19 test [15]. Contacts of confirmed patients were instructed to undergo COVID-19 testing and were subject to self-isolation, active surveillance, or simple testing depending on the level of contact. The results from all basic and in-depth epidemiological investigations conducted by public health centres were reported to the provincial office. In the event of a cluster infection involving several patients, a provincial epidemiological investigator created an infection schematic by referring to the in-depth epidemiological investigation results.

### Data source

The present study used a database that included information about confirmed COVID-19 cases based on positive PCR test results within Gangwon Province. The database included content verified by basic epidemiological investigations (demographic information, such as sex, age, and occupation; and epidemiological information, such as date of symptom onset, presence of symptoms, and Ct values) and information verified from the infection schematic created based on the in-depth epidemiological investigations (relationship between infector and infectee, suspected location of transmission occurrence, and suspected time and date of transmission).

### Study population

The present study analysed confirmed cases due to cluster infections among all confirmed COVID-19 cases in Gangwon Province that occurred between 22 February 2020 (the date on which the first case was reported) and 31 May 2021. Cluster infection was defined as ‘a group of 10 or more confirmed cases linked to the specific initiating person’.

Some clusters were not assessed through epidemiological investigations in Gangwon Province because all the patients in the cluster were not under the jurisdiction of public health centres of this province. Therefore, those cases were excluded from the analysis. Consequently, 75 cases in two clusters were excluded from the analysis due to the lack of an infection schematic given that the epidemiological investigations were not conducted in Gangwon Province. Of these, one cluster involved mass infection in a correctional facility and the other involved cases in which all confirmed patients came to Gangwon Province from another administrative district and stayed briefly at a single lodging property without any outside contact.

### Variables

This study characterised cluster infections with various ‘transmission routes’, which identify where/how the patient got infected. Transmission routes were characterized by measuring the relationship between the infector and the infectee and the location of occurrence. Both variables were characterized for all patients with confirmed COVID-19. The transmission routes were categorised as shown in Table 1. Five major categories of transmission routes were set according to the relationship between the infector and the infectee: (1) Cohabiting family members, (2) Social gatherings with non-cohabiting family members/relatives (3) Social gatherings with acquaintances, (4) Using (staying in) the same facility, and (5) Others. When cohabiting/non-cohabiting family members and acquaintances met together, we classified it as ‘Social gatherings with acquaintances’. ‘Using (staying in) the same facility’ included contact between individuals with unspecified relationships, contact between employees and customers, and contact between coworkers in the applicable facility, while social gatherings with non-cohabiting family members/relatives or acquaintances in the facility were excluded. The major category of ‘others’ comprised cases lacking an infection schematic due to difficulties in conducting the epidemiological investigations given that most of the confirmed patients were foreigners.

**Table 1.** Categories of transmission route

Major category	Subcategory
(1) Cohabiting family members	
(2) Social gatherings with non-cohabiting family members/relatives	Home
	Restaurants
	Other facilities except restaurants
	Unspecified location
(3) Social gatherings with acquaintances	Home
	Restaurants
	Other facilities except restaurants
	Unspecified location
(4) Using (staying in) the same facility	Bath houses
	Childcare
	Churches
	Colleges/universities
	Day care facilities
	Daycare centres and kindergartens
	Funeral homes
	Hobby schools
	Kitchens
	Learning centres
	Lodging
	Medical institutions (inpatient)
	Medical institutions (outpatient)
	Military bases
	Nightlife businesses
	Nursing homes
	Offices (contact between Worker-visitor / or among visitors)
	Offices (workers)
	Public institutions (contact between worker-visitor/ or among visitors)
	Public institutions (workers)
	Restaurants
	Schools
	Service businesses
	Shipping
	Sports facilities
	Stores
	Visiting nursing homes
(5) Others	

Among the five major categories of transmission routes, ‘social gatherings with non-cohabiting family members/relatives’ and ‘social gatherings with acquaintances’ were reclassified respectively into four sub-categories by the transmission location. ‘Using (staying in) the same facility’ was reclassified into 27 sub-categories by the transmission location.

### Data analysis

The *χ*^2^ test was used to analyse differences in sex, age, and occupational groups between the cluster infection cases and all confirmed cases and between cluster infection cases and non-cluster infection cases. Analyses were performed using SAS v9.4.

In the main analysis, transmission routes were characterized by referring to the infection schematic illustrating the link between the infector and infectee in each cluster. All infection schematics were completed by a designated provincial epidemiological investigator who had completed the epidemiological investigator training held by the KDCA. All infection schematics depicted the transmission of infection, starting from the index case (the first infected patient in a cluster recognized by a city/county public health centre) or the primary case (the first infected patient in a cluster who was actually the source of transmission). The index cases with unknown origin of infection were excluded from the major transmission route categories. They were classified as ‘Index cases with unknown origin source’, rather than in one of the five major categories.

**Fig. 1.**
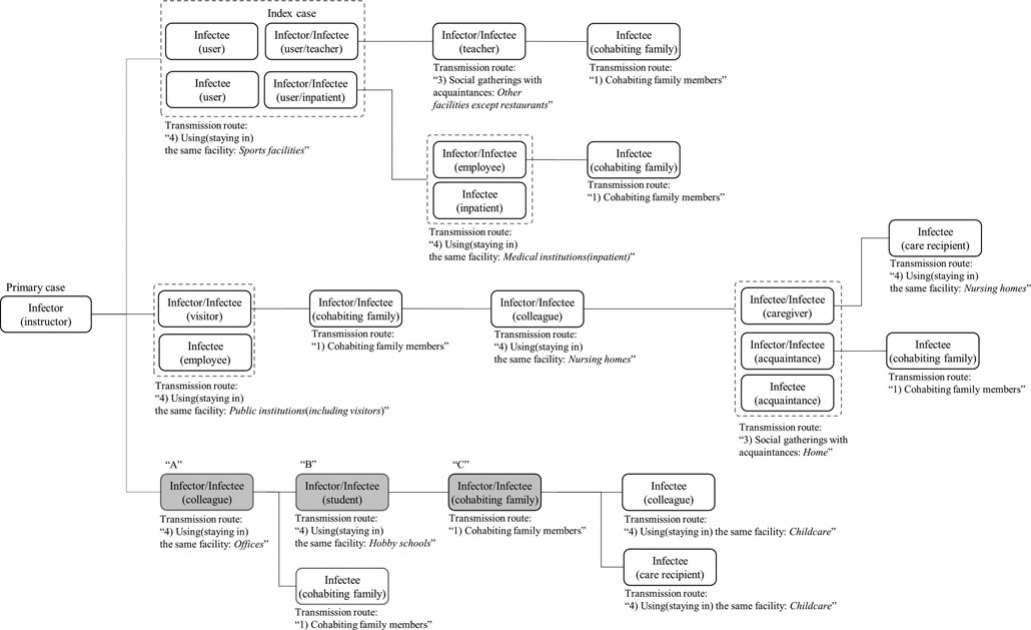
Example of characterisation of transmission route in an infection schematic. Part of an infection schematic of cluster infection. The location of transmission and the relationship between the infector and infectee were characterized by referring to an in-depth epidemiological investigation report of all confirmed cases.

To determine the final transmission routes, the relationship between the infector and infectee and the locations of suspected occurrence of transmissions within each infection schematic were characterized. Confirmed cases corresponding to each transmission route were then quantified under instructions of epidemiological investigator. The representative infection schematic of an actual cluster is presented in Figure 1. With the schematics, we determined the transmission route of each patient. For instance, in Figure 1, as A (infector) and B (infectee) were related to the specific place (hobby school), B's transmission route was classified as ‘using (staying in) the same facility (hobby school)’. Following A-B transmission, B (as an infector) and C (a new infectee) were related as cohabiting family members. Then C's transmission route was classified as ‘cohabiting family members’. Thus each person was related to one transmission route.

### Ethical considerations

As the present study used an existing database that included demographic information about each confirmed COVID-19 cases, it was approved by the Institutional Review Board (IRB) of Kangwon National University Hospital (IRB approval number: KNUH-2021-02-001).

## Results

### Cluster infection status

Since the first confirmed case of COVID-19 in Gangwon Province on 22 February 2020, there have been 3200 confirmed COVID-19 cases and 51 deaths as of 31 May 2021. Of these, 75 cases in 2 clusters were excluded as mentioned in the methods, leaving 3125 cases. A total of 61 COVID-19 clusters comprising 1741 (1741/3125; 55.7%) confirmed cases were analysed in Gangwon Province between 22 February 2020 and 31 May 2021.

**Fig. 2.**
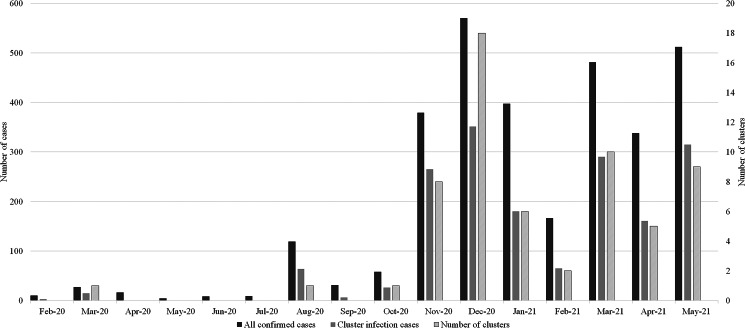
Monthly trends in numbers of clusters, confirmed cases, and cluster infection cases.

Figure 2 depicts the monthly trends (between 22 February 2020 and 31 May 2021) in the number of confirmed cases, the number of clusters, and the number of cluster infection cases. Since the number of cluster infection cases have a direct effect on the number of all confirmed cases, scatter plots between the number of cluster infection cases and the number of all confirmed cases, and between the number of clusters and the number of all confirmed cases showed positive correlation, respectively (Supplementary Fig. S1 and S2).

The number of confirmed cases, the duration of cluster infection, maximum generation in each cluster, and major transmission routes by cluster are presented in Supplementary Table S1. The median and range (minimum and maximum values) for the number of confirmed cases in the 61 clusters was 21 (10 and 129). The median with the range for infection duration within the clusters was 16 (3 and 44) days. The three clusters showed 7 transmission generations (Supplementary Table S1).

Analysis of the number of confirmed cases in the clusters revealed that the highest number of clusters (30/61; 49.2%) included 10–20 cases, whereas 8 of 61 clusters (13.1%) were large clusters with ⩾50 confirmed cases (Supplementary Table S2).

### Comparison between cluster infection cases and non-cluster infection cases

Sex, age, and occupational groups were compared between cluster and non-cluster infection cases in Gangwon Province (Table 2). In both groups, the number of infected men was higher than that of women, and the highest number of patients belonged to the 40–69 years age group. There was a significant difference in the age group between the two groups, but no significant differences in sex and occupational group.

**Table 2.** Demographic characteristics of all confirmed cases, cluster infection cases, and non-cluster infection cases

	All confirmed cases (%)	Cluster infection cases (%)	Non-cluster infection cases (%)	*P*-value*
Total	3125 (100)	1741 (100.0)	1384 (100)	
Sex				0.46
Male	1636 (52.4)	914 (52.5)	722 (52.2)	
Female	1489 (47.6)	827 (47.5)	662 (47.8)	
Age (years)				0.001
0–9	172 (5.5)	116 (6.7)	56 (4.0)	
10–19	232 (7.4)	145 (8.3)	87 (6.3)	
20–29	411 (13.2)	202 (11.6)	209 (15.1)	
30–39	394 (12.6)	226 (13.0)	168 (12.1)	
40–49	478 (15.3)	292 (16.8)	186 (13.4)	
50–59	508 (16.3)	299 (17.2)	209 (15.1)	
60–69	570 (18.2)	290 (16.7)	280 (20.2)	
70–79	222 (7.1)	97 (5.6)	125 (9.0)	
80–89	117 (3.7)	63 (3.6)	54 (3.9)	
⩾90	21 (0.7)	11 (0.6)	10 (0.7)	
Occupational groups				0.45
Managers	38 (1.2)	20 (1.1)	18 (1.3)	
Professionals and related workers	280 (9.0)	171 (9.8)	109 (7.9)	
Office workers	230 (7.4)	110 (6.3)	120 (8.7)	
Service workers	37 (9.8)	173 (9.9)	134 (9.7)	
Sales workers	198 (6.3)	103 (5.9)	95 (6.9)	
Skilled agricultural, forestry, and fishery workers	133 (4.3)	82 (4.7)	51 (3.7)	
Craft and related trades workers	50 (1.6)	27 (1.6)	23 (1.7)	
Plant and machine operators, and assemblers	72 (2.3)	38 (2.2)	34 (2.5)	
Elementary occupations	284 (9.1)	201 (11.5)	83 (6.0)	
Students, homemakers, armed forces, preschoolers, unknown	962 (30.8)	527 (30.3)	435 (31.4)	
Unemployed	571 (18.3)	289 (16.6)	282 (20.4)	

* *P*-value of homogeneity test (*χ*^2^ test) comparing cluster infection and non-cluster infection case groups.

### Transmission routes of cluster infections

The transmission routes of the 61 clusters (1741 cluster infection cases) were characterized and categorised according to the criteria presented in Table 1. The number and percentage of confirmed cases according to the five major categories of transmission routes are presented in Table 3. Among the five major categories of transmission routes, ‘using (staying in) the same facility’ was associated with the highest number of confirmed cases (*n* = 883 cases, 50.7%), followed by ‘cohabiting family members’ (*n* = 405, 23.3%), ‘social gatherings with acquaintances’ (*n* = 188, 10.8%), ‘other transmission routes’ (*n* = 121, 7.0%), and ‘social gatherings with non-cohabiting family members/relatives’ (*n* = 96, 5.5%).

**Table 3.** Number of cluster infection cases and clusters according to major categories of transmission route

Major categories of transmission route	Number of cluster infection cases (%)	Number of clusters (%)
Total	1741 (100.0)	61 (100.0)
(1) Cohabiting family members	405 (23.3)	58 (95.1)
(2) Social gatherings with non-cohabiting family members/relatives	96 (5.5)	26 (42.6)
(3) Social gatherings with acquaintances	188 (10.8)	44 (72.1)
(4) Using (staying in) the same facility	883 (50.7)	58 (95.1)
(5) Others	121 (7.0)	2 (3.3)
Index cases with unknown origin source	48 (2.8)	–

### Cohabiting family members

The number of confirmed cases of transmission in cohabiting family members was 23.3% of the total cases. However, transmission via cohabiting family members was identified in 58 of 61 clusters analysed (95.1%), and is equal to the identified cluster number of ‘using (staying in) the same facility.’

### Social gatherings with non-cohabiting family members/relatives or acquaintances

Transmissions through social gatherings with non-cohabiting family members/relatives and social gatherings with acquaintances were identified in 26 of 61 clusters (42.6%) and in 44 of 61 clusters (72.1%), respectively (Table 3). The highest number of confirmed cases (*n* = 82; Table 4) was attributed to social gatherings with acquaintances at home, which predominantly involved activities such as conversing or drinking tea or coffee at someone's home. Social gatherings with non-cohabiting family members/relatives in restaurants were identified in 3 clusters and 6 cases, whereas social gatherings with acquaintances in restaurants were identified in 12 clusters and 30 cases. Social gatherings with acquaintances in a facility, except restaurants, were associated with the highest number of clusters (27 clusters). There was no case attributed to social gatherings with non-cohabiting family members in an unspecified location.

**Table 4.** Totals by sub-categories of social gatherings with non-cohabiting family members/relatives or acquaintance

	Number of cluster infection cases (%)	Number of clusters (%)
Total social gathering	284 (100)	49 (100)
Social gatherings with non-cohabiting family members/relatives	96 (33.8)	26 (53.1)
Home	80 (28.0)	22 (44.9)
Restaurants	6 (2.1)	3 (6.1)
Other facilities except restaurants	10 (3.5)	4 (8.2)
Unspecified location	0 (0.0)	0 (0.0)
Social gatherings with acquaintances	188 (66.2)	45 (91.8)
Home	82 (28.9)	25 (51.0)
Restaurants	30 (10.6)	12 (24.5)
Other facilities except restaurants	55 (19.4)	27 (55.1)
Unspecified location	21 (7.4)	12 (24.5)

### Using (staying in) the same facility

Transmission due to using (staying in) the same facility was identified in 58 of 61 clusters (95.1%; Table 3), and this transmission route was associated with the highest number of confirmed cases (*n* = 883, 50.7%). Using (staying in) the same facility was further divided into 27 subcategories (Table 5). The transmission route with the highest number of confirmed cases was transmission within churches (*n* = 91 cases, 10.3%), followed by medical institutions (inpatient, *n* = 89, 10.1%), sports facilities (*n* = 86, 9.7%), military bases (*n* = 74, 8.4%), and offices (*n* = 68, 7.7%). Infection within offices were associated with the highest number of clusters (*n* = 23, 39.7%). The transmission route with the highest average number of confirmed cases per cluster was infection within nightlife businesses (65 confirmed cases/3 clusters, an average of 21.7 confirmed cases per cluster), followed by nursing homes (19.0), military bases (18.5), day-care facilities (16.0), medical institutions (inpatient, 11.1), and sports facilities (10.8). Additional descriptive statistics, including median values with the range, are presented in Supplementary Table S3.

**Table 5.** Totals by sub-categories of using (staying in) the same facility

Sub-categories	Number of cluster infection cases (%)	Number of clusters (%)
Total	883 (100)	58 (100)
Churches	91 (10.3)	12 (20.7)
Medical institutions (inpatient)	89 (10.1)	8 (13.8)
Sports facilities	86 (9.7)	8 (13.8)
Military bases	74 (8.4)	4 (6.9)
Offices	68 (7.7)	23 (39.7)
Nightlife businesses	65 (7.4)	3 (5.2)
Schools	64 (7.2)	10 (17.2)
Restaurants	42 (4.8)	14 (24.1)
Day-care centres and kindergarten	42 (4.8)	6 (10.3)
Service businesses	37 (4.2)	7 (12.1)
Hobby schools	37 (4.2)	9 (15.5)
Stores	37 (4.2)	7 (12.1)
Learning centres	21 (2.4)	8 (13.8)
Nursing homes	19 (2.2)	1 (1.7)
Public institutions (including visitors)	18 (2.0)	6 (10.3)
Bath houses	17 (1.9)	5 (8.6)
Day care facilities	16 (1.8)	1 (1.7)
Visiting nursing homes	9 (1.0)	6 (10.3)
Public institutions	9 (1.0)	3 (5.2)
Lodging facilities	9 (1.0)	5 (8.6)
Childcare	8 (0.9)	3 (5.2)
Kitchens	8 (0.9)	3 (5.2)
Funeral homes	6 (0.7)	1 (1.7)
Offices (including visitors)	5 (0.6)	4 (6.9)
Medical institutions (outpatient)	4 (0.5)	2 (3.4)
Colleges/universities	1 (0.1)	1 (1.7)
Shipping	1 (0.1)	1 (1.7)

### Other transmission routes

In two clusters, most cases involved foreign temporary workers (111/123, 90.2% and 10/16, 62.5%); as such, the infection schematic for these 121 patients could not be created. Accordingly, the transmission routes of these 121 patients were processed as other transmission routes. A proportion of these patients were co-habitants or colleagues, while others shared meals or participated in specific religious activities together. Multiple infections were associated with relationships that involved similar life patterns.

## Discussion

This study aimed to investigate the transmission routes and characteristics of cluster infections that occurred in Gangwon Province between the first confirmed case of COVID-19 on 22 February 2020 and 31 May 2021. Throughout the study period, 55.7% of confirmed COVID-19 cases in Gangwon Province were cluster infections. There were no differences in sex and occupation between the cluster infection cases and non-cluster infection cases. However, the number of older patients associated with the non-cluster infection group was more. Cluster analysis revealed that the transmission routes linked to the highest number of cases appeared in the order of using (staying in) the same facility, cohabiting family members, social gatherings with acquaintances, social gatherings with non-cohabiting family members/relatives.

### Transmission route: cohabiting family members

Transmission via cohabiting family members was identified in 95.1% of all clusters, and was identified as a transmission route in 58 of 61 clusters analysed, indicating the major influence of this transmission route. This result is in agreement with those of reports from outside of Korea [16–22]. In addition, a previous systematic review analysed 65 articles published between 1 January and 15 June 2020 and identified the major transmission routes in 108 cluster infections [5], which revealed that the most common type of cluster occurred in the order of family clusters, community transmission, nosocomial infection, transmission from gatherings, and transmission from transportation. The results of the present study were consistent with those of the previous systematic review in that cohabiting with family members accounted for the highest percentage of transmissions. However, cohabiting family members characteristically spend long periods in close contact with each other at home, which makes it challenging to adhere to disease control rules such as mask-wearing, hand hygiene, and social distancing. Consequently, it may be difficult to block transmission via cohabiting family members.

### Transmission route: social gatherings with non-cohabiting family members/relatives or with acquaintances

With regard to transmission through social gatherings with non-cohabiting family members/relatives or acquaintances, links with the sequence of ‘cohabiting family members’ → ‘social gatherings with non-cohabiting family members/relatives or acquaintances’ → ‘cohabiting family members’ were identified in seven clusters. This appeared to have a smaller influence as an infection link compared to that of using (staying in) the same facility. This could at least partly be due to the nationwide implementation of disease control policies by the South Korean government, which banned gatherings of five or more persons starting from 7 January 2021. No cases of simultaneous gatherings of five or more non-cohabiting family members/relatives or acquaintances among the seven clusters were identified.

### Transmission route: using (staying in) the same facility

The transmission route with the highest number of confirmed cases (883 cases) was ‘Using (staying in) the same facility’. Transmission due to ‘Using (staying in) the same facility’ exposes everyone concurrently staying in that facility to the risk of infection. Therefore, a higher number of people may be infected at once. Facilities with the highest number of confirmed cases appeared in the order of churches, medical institutions (inpatient), sports facilities, military bases, and offices while those with the highest number of clusters appeared in the order of offices, restaurants, churches, schools, and hobby schools.

It revealed that infection within churches was the most common, and this result was inconsistent with findings from Liu *et al*. indicating that medical institutions were the facility at which the most infections occurred [5]. This discrepancy could be due to the different regions at which the clusters occurred. Of the 65 articles analysed in the literature review, 46 articles examined clusters in China; among the clusters analysed in these 46 articles, only two clusters involved infection within religious facilities. In contrast, infection within churches was identified as the transmission route in 12 of 61 clusters analysed in the present study.

The above pattern was inconsistent with the frequency of use of these facilities by the general population. In 2015, the number of people in Korea who frequented churches, except catholic churches, was 9 675 761 (19.7%) [23], showing that the proportion of Christians, except Catholics, was relatively small. Furthermore, in another study in 2020, 31.5% of respondents answered ‘Do not exercise at all’ or ‘Almost do not exercise’ [24]. Thus, it is estimated that not many people visit the gym. On the other hand, our results indirectly indicate that places, such as churches and sports facilities, may be more vulnerable to infection. Some earlier studies have also found that many infections are transmitted at churches and sports facilities, as they are locations at which large crowds congregate indoors and engage in activities that produce droplets [25–30].

Facilities wherein many individuals stay and perform daily living activities were also highly susceptible to infection transmission. In particular, multiple cases of infections in medical institutions (inpatient) and military bases have been reported [31, 32]. In addition, infections within nightlife businesses, where people come in close contact with each other within a confined space with poor ventilation, have also been reported [33].

Among the clusters analysed, 13 clusters exhibited a link with the following sequence: cohabiting family members → using (staying in) the same facility → cohabiting family members. These findings suggest that using the same facility may be a link that leads to transmission from one family to another. Moreover, infection of two or more cohabiting family members via transmission due to using (staying in) the same facility was observed in 24 clusters. Therefore, while disease control among cohabiting family members may be difficult in practice, focusing on controlling transmission via the route of using (staying in) the same facility may play a major role in reducing the scale of clusters. In particular, it may be more effective to focus on disease control for facilities such as churches, medical institutions (inpatient), sports facilities, military bases, offices, schools, nightlife businesses, and restaurants, which were found to be vulnerable to transmission of infection in the present study.

### Strengths

This study has several strengths. First, this study analysed the transmission routes of all clusters linked to 10 or more cases that occurred in a single province, from the first confirmed case to May 2021. Although previous studies have analysed cluster infection cases, most of these studies selected specific clusters or conducted a systematic literature review of existing studies [5]. Second, this study divided and categorised transmission routes by the relationship between the infector and the infectee and the location of transmission occurrence. A study by Liu *et al*. combined existing studies and divided cluster transmission routes into nine categories by location [5]. However, in the present study, we divided transmission routes into 37 subcategories. Thus, we could reveal the vulnerable places for transmission in more detail. Third, this study characterized transmission routes through basic and in-depth epidemiological investigations of all confirmed clusters, and a formally trained epidemiological investigator reviewed all the categories of transmission routes. In-depth epidemiological investigation reports contained precise details about each confirmed patient's daily movements and contacts. Thus, we could characterise the relationship between the infector and the infectee rather than simply characterizing the place of transmission, and a review of the epidemiological investigator enabled the estimation of the transmission routes consistently and accurately.

### Limitations

The present study has several limitations. First, this study was unable to identify the transmission routes of small-scale infections involving less than 10 cases. Since 44.3% of the total confirmed cases during the study period in Gangwon province were in small groups of < 10 cases and/or were of unknown origin, further research is needed to investigate these. Second, due to the nature of epidemiological investigations using statements based on patient recall, characterization of the location of infection and the relationship between the infector and infectee was challenging for a proportion of cases. For these cases, the transmission routes could only be estimated based on the discretion of the epidemiological investigator.

## Conclusion

In conclusion, this study characterized the transmission routes of COVID-19 clusters that occurred in Gangwon Province between the first confirmed case and May 2021. Churches, medical institutions(inpatient), sports facilities, military bases, offices, nightlife businesses, schools, restaurants, day-care centres and kindergarten, and service businesses were identified as most vulnerable locations of infection transmission from using (staying in) the same facility. In settings where adhering to disease control guidelines, such as social distancing, may be difficult to do at home, the current findings highlight transmission routes that may be the most effective targets. Further studies should analyse infection clusters involving less than 10 cases in order to identify the transmission routes of small-scale infections.

## Data Availability

The data that support the findings of the present study are available on request from the corresponding author. The data are not publicly available due to privacy and legal restrictions.

## Supplementary material

For supplementary material accompanying this paper visit https://doi.org/10.1017/S0950268821002788. click here to view supplementary material
