# Supplementary material for: Characteristics of transmission routes of COVID-19 cluster infections in Gangwon Province, Korea
Source: Epidemiol Infect. 2022 Jan 7;150:e19. doi: 10.1017/S0950268821002788 (PMC8770846; doi:10.1017/S0950268821002788)
Supplement: Supplementary file 1 [file hygsup.zip › S0950268821002788sup001.docx]

*Epidemiology and Infection*

Characteristics of Transmission Routes of COVID-19 Cluster Infections in Gangwon Province, Korea

Chaeyun Lim^1^, Youngju Nam^1^, Won Sup Oh^1,2^, Sugeun Ham^1^, Eunmi Kim^1^, Myeonggi Kim^1^, Saerom Kim^1^, Yeojin Kim^1^ and Seungmin Jeong^1,3*^

^1.^ Gangwon Centre for Infectious Diseases (affiliated to Korea Disease Control and Prevention Agency and Gangwon Provincial Office), Gangwon, Korea

^2.^ Division of Infectious Diseases, Kangwon National University Hospital, Gangwon, Korea

^3.^ Department of Preventive Medicine, Kangwon National University Hospital, Gangwon, Korea

*Correspondence to: Associate Professor Seungmin Jeong (Email: seungminjeong226@gmail.com)

Supplementary Material

Supplementary Table S1. Cluster infections occurred in Gangwon Province

|  | Cluster name | Number of confirmed cases (patients) | Duration of infection  (days) | Maximum  generation | Major transmission route |
| --- | --- | --- | --- | --- | --- |
| 1 | Cheorwon cluster | 129 | 44 | 7 | Military base and cohabiting family members |
| 2 | Donghae Hospital | 31 | 38 | 2 | Medical institutions (inpatient) |
| 3 | Gangneung foreign temporary workers | 123 | 36 | 4 | Same living space |
| 4 | Cheorwon nursing care facility | 44 | 30 | 5 | Cohabiting family members and day care facilities |
| 5 | Donghae Elementary School | 64 | 29 | 4 | Cohabiting family members and school |
| 6 | Sokcho Medical Centre | 28 | 29 | 5 | Cohabiting family members and medical institution (inpatient) |
| 7 | Wonju church 2 | 20 | 26 | 3 | Cohabiting family members, social gatherings with acquaintances, and church |
| 8 | Wonju ophthalmology clinic | 19 | 24 | 5 | Cohabiting family members and medical institution (outpatient) |
| 9 | Sokcho nursing hospital | 29 | 23 | 2 | Medical institution (inpatient) |
| 10 | Chuncheon church | 19 | 22 | 4 | Cohabiting family members and church |
| 11 | Entertainment businesses in Chuncheon | 49 | 21 | 6 | Cohabiting family members and entertainment business |
| 12 | Wonju church | 43 | 21 | 4 | Cohabiting family members and church |
| 13 | Pyeongchang resort | 27 | 21 | 5 | Service business and school |
| 14 | Hwacheon cluster | 12 | 21 | 3 | Cohabiting family members and social gatherings with acquaintances |
| 15 | Social gatherings with acquaintances in Gangneung | 56 | 20 | 7 | Cohabiting family members, sales outlet, and social gatherings with acquaintances |
| 16 | Sokcho sports facility | 24 | 20 | 6 | Cohabiting family members and sports facility |
| 17 | Pyeongchang funeral home | 24 | 20 | 5 | Cohabiting family members and social gatherings with acquaintances |
| 18 | Jeongseon church | 48 | 19 | 6 | Cohabiting family members and church |
| 19 | Pyeongchang resort employees | 35 | 19 | 6 | Cohabiting family members and office |
| 20 | Hongcheon day workers | 23 | 19 | 5 | Cohabiting family members and service business |
| 21 | Wonju indoor sports facility | 70 | 18 | 7 | Cohabiting family members and sports facility |
| 22 | Sokcho indoor sports facility | 53 | 18 | 5 | Cohabiting family members and sports facility |
| 23 | Wonju cluster | 29 | 18 | 6 | Cohabiting family members and family gatherings |
| 24 | Donghae Multicultural Family Support Centre | 18 | 18 | 4 | Cohabiting family members and office |
| 25 | Donghae cluster | 14 | 18 | 4 | Cohabiting family members and office |
| 26 | Sokcho childcare centre | 52 | 17 | 6 | Cohabiting family members and daycare centre/kindergartens |
| 27 | Cheorwon tennis centre | 21 | 17 | 5 | Cohabiting family members and sports facility |
| 28 | Wonju music facility | 13 | 17 | 4 | Cohabiting family members and hobby school |
| 29 | Donghae church | 12 | 17 | 3 | Church and bath house |
| 30 | Chuncheon children centre | 10 | 17 | 3 | Public institution |
| 31 | Entertainment businesses in Wonju | 51 | 16 | 5 | Cohabiting family members and entertainment business |
| 32 | Cheorwon military base | 28 | 16 | 3 | Military base |
| 33 | Donghae supermarket | 25 | 16 | 4 | Cohabiting family members and store |
| 34 | Chuncheon National University | 23 | 16 | 4 | Cohabiting family members and kitchen |
| 35 | Gangneung Culture Centre | 22 | 16 | 4 | Public institution (including visitors) and cohabiting family members |
| 36 | Yeongwol cluster | 21 | 16 | 5 | Cohabiting family members and school |
| 37 | Wonju health centre | 20 | 16 | 5 | Cohabiting family members and sports facility |
| 38 | Wonju church 3 | 19 | 16 | 4 | Cohabiting family members and church |
| 39 | Chuncheon learning centre | 18 | 16 | 4 | Cohabiting family members and learning centre |
| 40 | Yanggu cluster | 12 | 16 | 4 | Cohabiting family member and family gatherings |
| 41 | Inje multi-level marketing | 32 | 15 | 5 | Cohabiting family members and social gatherings with acquaintances |
| 42 | Wonju choir | 26 | 15 | 4 | Cohabiting family members and hobby school |
| 43 | Wonju medical device demonstration store | 25 | 15 | 4 | Cohabiting family members and store |
| 44 | Jeongseon hospital visit | 15 | 15 | 3 | Cohabiting family members and social gatherings with acquaintances |
| 45 | Gangneung health centre | 17 | 14 | 4 | Cohabiting family members and sports facility |
| 46 | Wonju cluster 2 | 14 | 14 | 3 | Cohabiting family members and family gatherings |
| 47 | Hoengseong Hospital | 11 | 14 | 4 | Cohabiting family members and medical institution (inpatient) |
| 48 | Shincheonji | 18 | 13 | 4 | Cohabiting family members and church |
| 49 | Donghae Coast Guard | 13 | 13 | 3 | Cohabiting family members, office, and social gatherings with acquaintances |
| 50 | Donghae restaurant | 47 | 12 | 4 | Cohabiting family members and regular restaurants and bar |
| 51 | Gangneung bath house | 15 | 12 | 3 | Cohabiting family members and bath house |
| 52 | Donghae hospital 2 | 17 | 11 | 4 | Cohabiting family members, medical institution (inpatient), and store |
| 53 | Wonju childcare centre | 15 | 11 | 4 | Cohabiting family members and childcare centres |
| 54 | Gangneung birthday party | 11 | 11 | 5 | Cohabiting family members and family gatherings |
| 55 | Donghae cement company | 11 | 11 | 2 | Office |
| 56 | Gangneung Korean confectionery | 14 | 10 | 4 | Cohabiting family members and social gatherings with acquaintances |
| 57 | Donghae construction company | 11 | 4 | 3 | Cohabiting family members and office |
| 58 | Donghae cluster 2 | 10 | 4 | 3 | Cohabiting family members and family gatherings |
| 59 | Foreign temporary workers in Pyeongchang | 16 | 3 | 2 | Foreign temporary workers |
| 60 | Family gathering in Pyeongchang | 15 | 3 | 4 | Cohabiting family members and family gatherings |
| 61 | Taebaek restaurant | 10 | 3 | 3 | Cohabiting family members and regular restaurant and bar |

Supplementary Table S2. Cluster categories according to number of confirmed cases

| Number of confirmed cases | Number of clusters (%) | |
| --- | --- | --- |
| Total | 61 | (100) |
| 10-20 | 30 | (49.2) |
| 21-30 | 15 | (24.6) |
| 31-40 | 3 | (4.9) |
| 41-50 | 5 | (8.2) |
| ≥ 51 | 8 | (13.1) |

Supplementary Table S3. Descriptive statistics of cluster infection by sub-categories of using (staying in) the same facility

| Sub-categories | Number of cluster infection cases | Number of clusters | Number of cases per cluster | Median  number of cases | Minimum  number of cases | Maximum  number of cases |
| --- | --- | --- | --- | --- | --- | --- |
| Nightlife businesses | 65 | 3 | 21.7 | 30 | 1 | 34 |
| Nursing homes | 19 | 1 | 19.0 | 19 | 19 | 19 |
| Military bases | 74 | 4 | 18.5 | 14 | 1 | 45 |
| Day-care facilities | 16 | 1 | 16.0 | 16 | 16 | 16 |
| Medical institutions (inpatient) | 89 | 8 | 11.1 | 6 | 2 | 27 |
| Sports facilities | 86 | 8 | 10.8 | 7 | 1 | 39 |
| Churches | 91 | 12 | 7.6 | 5 | 1 | 30 |
| Restaurants | 42 | 6 | 7.0 | 3 | 1 | 26 |
| Schools | 64 | 10 | 6.4 | 3 | 1 | 31 |
| Funeral homes | 6 | 1 | 6.0 | 6 | 6 | 6 |
| Service businesses | 37 | 7 | 5.3 | 6 | 1 | 9 |
| Hobby schools | 37 | 7 | 5.3 | 2 | 1 | 17 |
| Stores | 37 | 9 | 4.1 | 2 | 1 | 18 |
| Bath houses | 17 | 5 | 3.4 | 2 | 1 | 8 |
| Day-care centres and kindergarten | 42 | 14 | 3.0 | 2 | 1 | 13 |
| Public institutions (including visitors) | 18 | 6 | 3.0 | 3 | 1 | 5 |
| Visiting nursing homes | 9 | 3 | 3.0 | 1 | 1 | 7 |
| Offices | 68 | 23 | 3.0 | 2 | 1 | 9 |
| Childcare | 8 | 3 | 2.7 | 1 | 1 | 6 |
| Kitchens | 8 | 3 | 2.7 | 1 | 1 | 6 |
| Learning centres | 21 | 8 | 2.6 | 2 | 1 | 8 |
| Medical institutions (outpatient) | 4 | 2 | 2.0 | 2 | 1 | 3 |
| Public institutions | 9 | 5 | 1.8 | 2 | 1 | 3 |
| Lodging facilities | 9 | 6 | 1.5 | 1 | 1 | 3 |
| Offices (including visitors) | 5 | 4 | 1.3 | 1 | 1 | 2 |
| Colleges/universities | 1 | 1 | 1.0 | 1 | 1 | 1 |
| Shipping | 1 | 1 | 1.0 | 1 | 1 | 1 |

Supplementary Figure S1. Scatter plot of number of clusters and number of all confirmed cases per month

Supplementary Figure S2. Scatter plot of number of cluster infection cases and number of all confirmed cases per month
