# Supplementary figures and images for: Characteristics of transmission routes of COVID-19 cluster infections in Gangwon Province, Korea
Source: Epidemiol Infect. 2022 Jan 7;150:e19. doi: 10.1017/S0950268821002788 (PMC8770846; doi:10.1017/S0950268821002788)

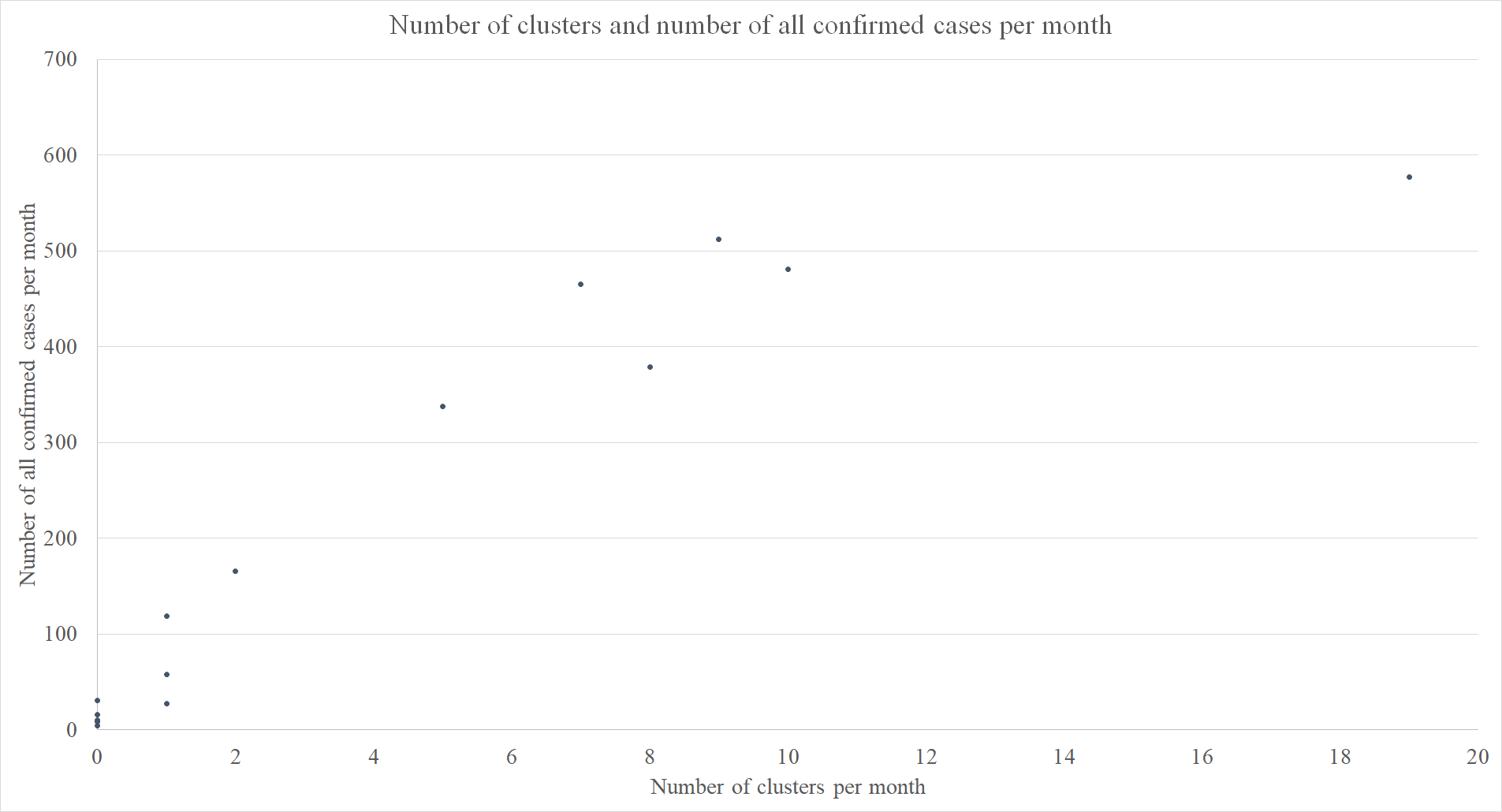

Supplement: Supplementary file 1 [file hygsup.zip › S0950268821002788sup002.png]

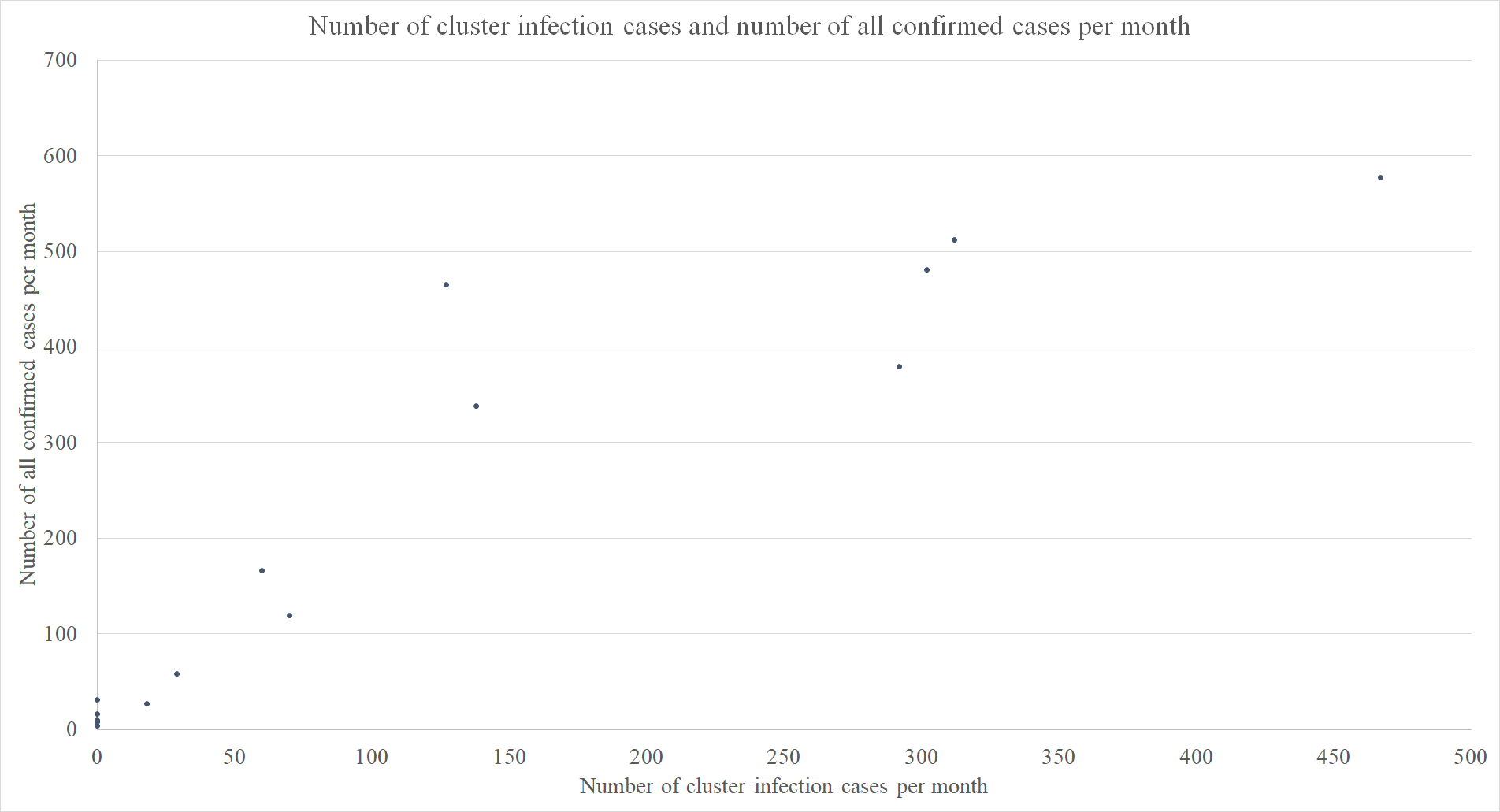

Supplement: Supplementary file 1 [file hygsup.zip › S0950268821002788sup003.png]
